# Multiple functions of SWI/SNF chromatin remodeling complex in plant-pathogen interactions

**DOI:** 10.1007/s44154-021-00019-w

**Published:** 2021-12-09

**Authors:** Yunqing Jian, Won-Bo Shim, Zhonghua Ma

**Affiliations:** 1 State Key Laboratory of Rice Biology, and Key Laboratory of Molecular Biology of Crop Pathogens and Insects, Institute of Biotechnology, Zhejiang University, Hangzhou, China; 2 Department of Plant Pathology and Microbiology, Texas A&M University, College Station, TX USA

**Keywords:** Chromatin remodeling, SWI/SNF complex, Transcription, Splicing, DNA damage repair, Plant-pathogen interaction

## Abstract

The SWI/SNF chromatin remodeling complex utilizes the energy of ATP hydrolysis to facilitate chromatin access and plays essential roles in DNA-based events. Studies in animals, plants and fungi have uncovered sophisticated regulatory mechanisms of this complex that govern development and various stress responses. In this review, we summarize the composition of SWI/SNF complex in eukaryotes and discuss multiple functions of the SWI/SNF complex in regulating gene transcription, mRNA splicing, and DNA damage response. Our review further highlights the importance of SWI/SNF complex in regulating plant immunity responses and fungal pathogenesis. Finally, the potentials in exploiting chromatin remodeling for management of crop disease are presented.

## Introduction

Eukaryotic genome are organized and compacted by 146 base pairs of DNA wrapped around a histone octamer, forming structures named nucleosomes, which enable long DNA strands to precisely fit into the nucleus (Mittal & Roberts, 2020; Ojolo et al. 2018). Within the nucleus, DNA strands are highly folded, constrained, and compacted into higher order chromatin structures, and the dynamic regulation of chromatin can ensure the appropriate timing, location and sequence of cellular DNA-based events (Luger et al. 2012; Roberts & Orkin, 2004). Therefore, mechanisms that govern chromatin dynamics are integral components of eukaryotic gene regulation, that include covalent histones, DNA modifications and ATP-dependent chromatin remodeling complexes (Centore et al. 2020; Roberts & Orkin, 2004).

Chromatin remodelers utilize the energy derived from ATP (adenosine triphosphate) hydrolysis to move, destabilize, eject, or restructure nucleosomes, thus modulating the access of transcription machinery to DNA (Becker & Hörz, 2002; Hohmann & Vakoc, 2014). The ATP-dependent chromatin remodeling complex can be divided into four distinct subfamilies: SWI/SNF, ISWI, CHD and INO80. These subfamilies share a conserved ATPase domain but functions in a largely non-redundant manner to govern discrete biological processes, such as transcriptional regulation, DNA replication, DNA repair, homologous recombination and chromosomal segregation (Clapier & Cairns, 2009; Hohmann & Vakoc, 2014; Masliah-Planchon et al. 2015). Among the four chromatin remodeling complex subfamilies, the SWI/SNF complex is evolutionarily conserved and was first discovered through genetic screens and biochemical purification in budding yeast *Saccharomyces cerevisiae* (Mittal & Roberts, 2020; Neigeborn & Carlson, 1984). Notably, the SWI/SNF complex is the most strongly associated regulator of chromatin access (Clapier et al. 2017; Euskirchen et al. 2012). Many studies have shown that the SWI/SNF complex regulates various stress responses and developmental pathways in budding yeast, *Drosophila*, and human through precise control of gene expression (Kasten et al. 2011; Kwon & Wagner, 2007). In this review, we highlight key functions and regulatory mechanisms of this complex in regulating plant-pathogen interactions.

## Compositions of the SWI/SNF complex

The conserved components of SWI/SNF complex were characterized in several eukaryotic organisms including *S. cerevisiae*, Arabidopsis and human (Table 1). All core and actin-related subunits, as well as the transcription associated component Swp73/Snf12, are well conserved in various eukaryotes. However, each organism uniquely constructs distinct SWI/SNF complex using both the conserved components and unique subunits. Even though the complex normally contains 9–12 subunits (Roberts & Orkin, 2004; Smith et al. 2003), only five of these, i.e. Swi2/Snf2 (the ATPase subunit), Snf5, Swi3, Arp7 and Arp9, are comparable or almost comparable to the entire complex when we take budding yeast as an example (Phelan et al. 1999; Roberts & Orkin, 2004; Zhang et al. 2018). Studies on the subunit architecture in *S. cerevisiae* revealed that the loss of Arp7 or Arp9 disrupts the catalytic core of SWI/SNF (Zhang et al. 2018). While in the absence of Snf5, the catalytic ternary complex Snf2-Arp7-Arp9 could be fully detached, suggesting its role in coordinating the distinct modules in SWI/SNF (Dutta et al. 2017; Zhang et al. 2018). In addition, the locations of Snf5, Swp82 and Swi1 indicate that these subunits are associated with the binding of the complex with transcription factors (Prochasson et al. 2003; Zhang et al. 2018).

**Table 1:** The components of SWI/SNF complex in various eukaryotic organisms

***Saccharomyces cerevisiae***	***Drosophila*** ***melanogaster***	***Homo sapiens***	***Arabidopsis thaliana***	Function
Swi2/Snf2	BRM	BRG1, BRM	BRM, SYD, CHR12, CHR23	Core subunit, ATPase activity
Snf5	SNR1/BAP45	SNF5/INI1	BSH	Core subunit
Swi3	MOR/BAP155	BAF155 BAF170	AtSwi3A AtSwi3B AtSwi3C AtSwi3D	Core subunit
Arp9 Arp7	BAP55 BAP47	BAF53A BAF53B	AtArp7 AtArp4	Actin related
Swp73/Snf12	BAP60	BAF60A BAF60B BAF60C	CHC1, CHC2	Transcription associated
Swi1/Adr6	OSA	BAF250/hOSA1		Prion protein
	Polybromo	Polybromo		Transcription associated
	β-actin	β-actin	β-actin	Actin related
	BAP111/dalao	BAF57		
Swp82 Snf6 Taf14 Snf11 Rtt102				Specific subunits for yeast

**Fig. 1:**
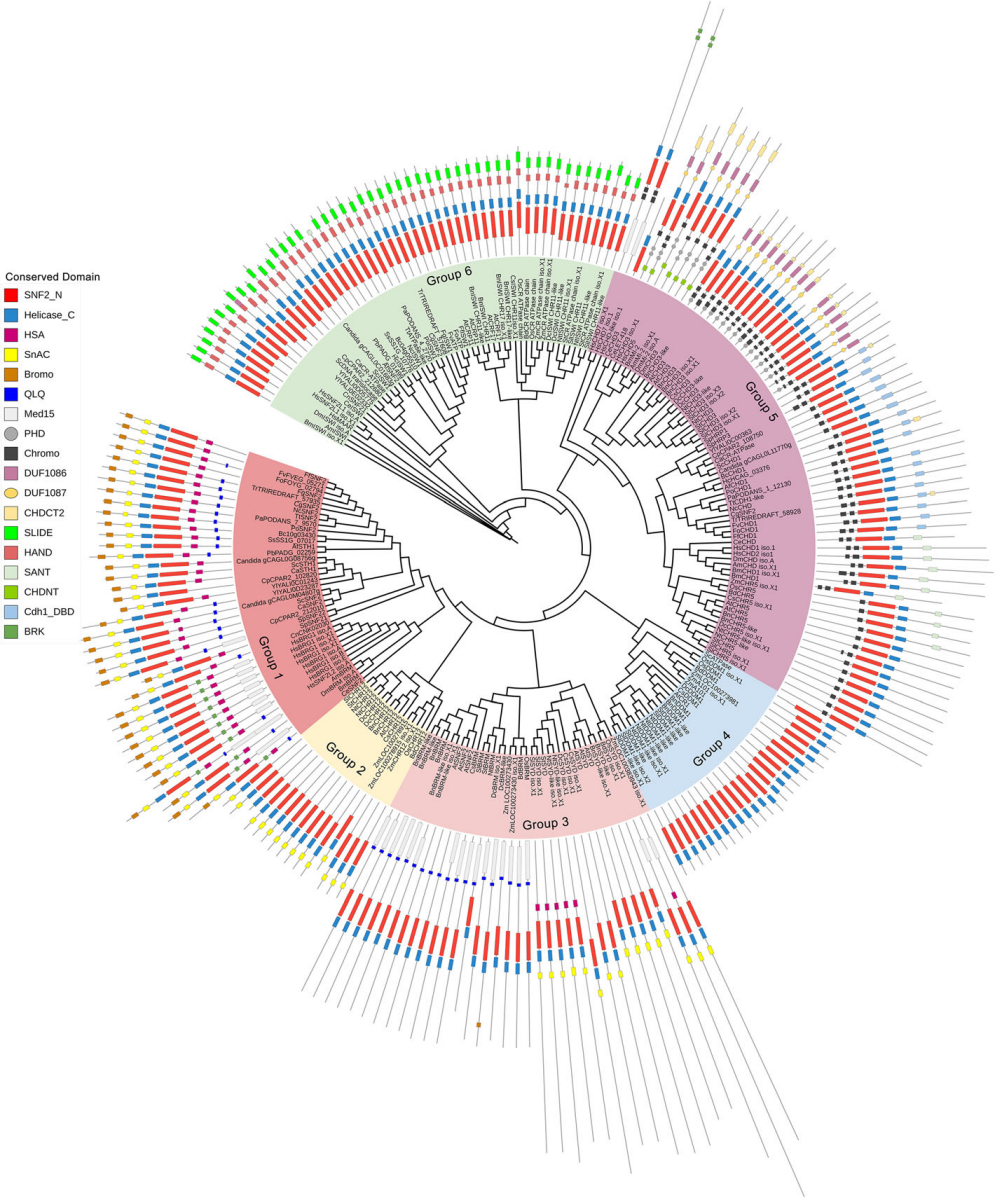
Phylogenetic tree of ATPases in animals, plants, and fungi. The sequence of ScSnf2 (YOR290C) was used as a bait to elicit all sequences with high homology and annotation, and the threshold was set to e-value = 1e^− 10^. A maximum-likelihood phylogenetic tree was constructed with sequences of ATPases or their homologs. All predicted ATPases were attributed to six groups (Group 1–6) based on the composition of domains. Different groups and conserved domains of ATPases were indicated by different colors. Am: *Apis mellifera*; At: *Arabidopsis thaliana*; Af: *Aspergillus fumigatus*; Bm: *Bombyx mori*; Bc: *Botrytis cinerea*; Bd: *Brachypodium distachyon*; Bn: *Brassica napus*; Ce: *Caenorhabditis elegans*; Ca: *Candida albicans*; Candida g: *Candida glabrata*; Cp: *Candida parapsilosis*; Cg: *Colletotrichum graminicola*; Cn: *Cryptococcus neoformans*; Cs: *Cucumis sativus*; Dc: *Daucus carota*; Dm: *Drosophila melanogaster*; Ff: *Fusarium fujikuroi*; Fg: *Fusarium graminearum*; Fo: *Fusarium oxysporum*; Fv: *Fusarium verticillioides*; Hc: *Histoplasma capsulatum*; Hs: *Homo sapiens*; Nc: *Neurospora crassa*; Nt: *Nicotiana tabacum*; Os: *Oryza sativa*; Pb: *Paracoccidioides brasiliensis*; Pa: *Podospora anserine*; Po: *Pyricularia oryzae*; Sc: *Saccharomyces cerevisiae*; Sp: *Schizosaccharomyces pombe*; Ss: *Sclerotinia sclerotiorum*; Sl: *Solanum lycopersicum*; St: *Solanum tuberosum*; Tt: *Thermothelomyces thermophiles*; Tr: *Trichoderma reesei*; Yl: *Yarrowia lipolytica*; Zm: *Zea mays*

The number of conserved components also varies from species to species, especially the most vital components associated with ATPase activity (Table 1). This finding prompted us to construct a phylogenetic tree to characterize the homologs of Snf2 and their functional domains in animals, plants and fungi. As shown in Fig. 1, the predicted ATPases were clustered into six groups: Group 1, SNF2 and BRG1; Group 2, CHR12; Group 3, BRM and SYD; Group 4, DDM1; Group 5, CHD1; and Group 6, ISWI. All group members contain two highly conserved domains, SNF2_N and Helicase_C, while different groups having unique domains. For instance, only group 1 members contain HSA and bromo domain, and the bromo domain is capable of binding to acetylated histones, which suggests that members of group 1 may function as reader proteins of acetylation (Jarończyk et al. 2021). SnAC domain is distributed in groups 1 and 2, and the SYD domain in members of group 3. The QLQ domain is present only in groups 1 and 3. Group 4 members have the shortest protein length, and they only contain the two conserved domains SNF2_N and Helicase_C. Interestingly, almost all members of group 4 originate from plants, and most of them are DDM1 or DDM1-like proteins. These proteins play a role in maintaining DNA methylation even though they have no methyltransferase activity (Ramirez-Prado et al. 2018; Vongs et al. 1993). Another noticeable finding is that the chromo domain is strictly distributed within the group 5. This domain is implicated in the binding of the proteins that are found to methylate histone tails and RNAs (Lu et al. 2020; Pray-Grant et al. 2005). It is noteworthy that the CHD3 members of group 5 own three extra domains, i.e.*,* PHD, DUF1086, and DUF1087. Among them, the PHD is considered as a histone code reader (Wang et al. 2021). In addition, the components of group 6 also contain two distinct domains SLIDE and HAND that are associated with extra-nucleosomal DNA and the entry site of nucleosomes (Dang & Bartholomew, 2007).

## Functional mechanisms of the SWI/SNF complex

### Regulatory roles of the SWI/SNF complex in gene transcription

The SWI/SNF remodelers are key regulators of nucleosome positioning, which typically controls chromatin accessibility and binding sites for transcriptional machinery at the gene promoters or enhancers, thus leading to either gene activation or repression (Clapier et al. 2017; Euskirchen et al. 2012). Genome-wide analysis showed that approximately 5% of genes are regulated by the SWI/SNF at the transcription level in the budding yeast (Sudarsanam et al. 2000), flies (Zraly et al. 2006), as well as mice (Gresh et al. 2005). However, due to the low intracellular level and the lack of intrinsic DNA binding specificity of the SWI/SNF complexes, they need to be guided by gene-specific transcriptional regulators, covalent histone modifiers or long noncoding RNAs (lncRNAs) to facilitate specific gene loci targeting in various organisms (Peterson & Workman, 2000; Sanz et al. 2012).

In *A. thaliana*, the physical interaction of SWI/SNF subunits with different proteins governs a wide range of developmental processes, such as embryo, leaf and flower organ development, response to plant hormones, and abiotic stresses (Ramirez-Prado & Benhamed, 2021; Reyes, 2014). A plant-unique H3K27 demethylase REF6 targets the CTCTGYTY motif-containing genomic loci through its zinc-finger (ZnF) domains and further facilitates recruitment of the BRM complex (Li et al. 2016). The Arabidopsis SYD (a homolog of the yeast Snf2p ATPase) can act as a transcriptional repressor of the meristem identity switch in the floral transition via interacting with and altering activity of the plant-specific transcriptional activator LEAFY (Wagner & Meyerowitz, 2002). Upon auxin sensing, the MONOPTEROS transcription factor recruits the BRM complex to increase DNA accessibility for induction of key regulators of flower primordium initiation (Wu et al. 2015). In Arabidopsis, some lncRNAs are produced by a specialized RNA Polymerase V (Pol V). The Pol V-produced lncRNAs can be associated with IDN2, a lncRNA-binding protein. Interestingly, SWI3B, an essential subunit of the BRM complex physically interacts with DN2, and subsequently contributes to lncRNAs-mediated transcriptional silencing (Zhu et al. 2013).

The fungal SWI/SNF complexes work in concert with various transcription factors and covalent histone modifiers to facilitate maintaining proper chromatin accessibility landscapes during fungal development and stress responsive processes (Table 2), which is consistent with those in plants. When we look at yeast as an example, the SWI/SNF complex functions together with regulatory protein Yap8 to mediate transcriptional activation of *ACR2* and *ACR3* in response to arsenic stress in *S. cerevisiae* (Menezes et al. 2017). While transcription factor Cha4 recruits SWI/SNF to initiate *SRG1* transcription by remodeling the two nucleosomes located at the *SRG1* transcription start site when serine is available to the cells (Hainer et al. 2011). When yeast encounters cell wall stress, the downstream transcription factor of cell wall integrity pathway Rlm1 physically interacts with SWI/SNF to direct its association to target promoters (Sanz et al. 2012). Moreover, several reports showed that the SWI/SNF complex is recruited by multiple transcription factors. For instance, Adr1 and Cat8 recruits SWI/SNF complex upon glucose repression (Biddick et al. 2008), whereas two classes of transcriptional activators HSF and Msn2/4 associate with this complex to modulate chromatin disassembly at heat shock gene promoters (Erkina et al. 2008; Shivaswamy & Iyer, 2008). One interesting report demonstrated that the yeast repressor Sko1 recruits Cyc8(Ssn6)-Tup1 corepressor complex to regulate transcription of genes that are induced upon hyperosmotic stress. During this process, the MAP kinase Hog1 associates with target promoters, phosphorylates Sko1, and converts Sko1 into a transcriptional activator. Subsequently, the formation of Sko1/Hog1/Tup1 ternary transcription activator complex is important for SWI/SNF recruitment during the transcriptional induction process (Proft & Struhl, 2002). Additionally, histone modifiers can also mediate the recruitment of SWI/SNF complex. As yeast cells progress through the cell cycle, the activator Swi5 enters into nuclei at the end of anaphase, which recruits both SWI/SNF and Spt-Ada-Gcn5-Acetyltransferase (SAGA) complexes to the *HO* endonuclease promoter (Cosma et al. 2016). Nucleosome arrays provide a functional link between histone acetylation and the SWI/SNF complex, and the retention of SWI/SNF is mediated by histone acetylation (Hassan et al. 2001). Further studies suggest that SWI/SNF preferentially displaces acetylated histones from the array relative to total histones, and the acetyl-lysine binding domain, Swi2/Snf2 bromodomain, plays a vital role in this process (Chandy et al. 2006; Mitra et al. 2006). Upon phosphate depletion, chaperone anti-silencing function 1 (Asf1) recruits SWI/SNF complex to promote chromatin disassembly at the yeast *PHO5* promoter (Adkins et al. 2007).

**Table 2:** Recruiters of SWI/SNF complex in modulating fungal growth and stress responses

Number	Fungal species	Recruiter	Target gene	Process	Reference(s)
1	*Saccharomyces cerevisiae*	Yap8	*ACR2* *ACR3*	Arsenic stress	(Menezes et al. 2017)
2	*S. cerevisiae*	Cha4	*SRG1*	Serine available	(Hainer et al. 2011)
3	*S. cerevisiae*	Swi5	*HO*	Cell cycle	(Cosma et al. 2016)
4	*S. cerevisiae*	Sko1/Hog1/Tup1 ternary	*GRE2* *AHP1* *HAL1*	Hyperosmotic stress	(Proft & Struhl, 2002)
5	*S. cerevisiae*	Rlm1	*MLP1* *KDX1*	Cell wall stress	(Sanz et al. 2012)
6	*S. cerevisiae*	Asf1	*PHO5*	Phosphate depletion	(Adkins et al. 2007)
7	*S. cerevisiae*	Adr1 Cat8	*ADH2* *FBP1*	Glucose repression	(Biddick et al. 2008)
8	*S. cerevisiae*	HSF Msn2/4	*HSP12* *HSP82* *SSA4*	Heat shock	(Erkina et al. 2008; Shivaswamy & Iyer, 2008)
9	*S. cerevisiae*	Gal4, VP16, Gcn4, Hap4	–	–	(Yudkovsky et al. 1999)
10	*S. cerevisiae*	SAGA NuA4	–	–	(Hassan et al. 2001)
11	*Neurospora*	WC-1	*FRQ*	Circadian cycle	(Wang et al. 2014)
12	*Cryptococcus neoformans*	Znf2	*ZNF2*	Yeast-to-hypha differentiation	(Lin & Zhao, 2019)
13	*Candida* *albicans*	Mrr1	*MDR1*	Fluconazole resistance	(Liu & Myers, 2017)
14	*Trichoderma* *reesei*	XYR1	*QM9414*	Cellulolytic response	(Cao et al. 2019)
15	*Fusarium* *graminearum*	FgAreB	*FHB1* *FHB2* *GSNOR* *NOR*	Nitrosative stress	(Jian et al. 2021)
16	*F.* *graminearum*	FgSR	*CYP51A* *CYP51B* *CYP51C*	Tebuconazole/ phytoalexin stress	(Liu et al. 2019)

### Involvement of the SWI/SNF complex in mRNA splicing

In higher eukaryotes, mRNA splicing is important for controlling both qualitative and quantitative aspects of gene expression. Moreover, the alternative splicing from a single gene can generate multiple functionally distinct protein isoforms, which greatly expands the genetic plasticity of an organism and has emerged as a vital layer of gene regulation in response to diverse stresses (Black, 2000; Blencowe, 2006; Pleiss et al. 2007). Changes in chromatin structure have been shown to affect mRNA splicing (Allemand et al. 2016; Kornblihtt, 2006). The genome-wide mapping of nucleosome positioning from different organisms shows that nucleosomes are particularly enriched at intron-exon junctions (Luco et al. 2011), which is evolutionarily conserved from plants to mammals, suggesting an essential role of nucleosome positioning in exon definition (Luco et al. 2011; Schwartz et al. 2009).

The SWI/SNF has been reported to interact with Pol II (Kornblihtt, 2006), thus influencing the efficiency of splicing (Allemand et al. 2016). For example, the accumulation of phosphorylated RNA Pol II in a central block of alternative exon of the CD44 gene in human cells can be caused by the overexpression of Brm, the core ATPase of SWI/SNF complex, which leads to the increased accumulation of mature mRNA (Batsché et al. 2006). Furthermore, the SWI/SNF complex also regulates mRNA splicing by modulating the transcription elongation rate of RNA Pol II through its subunit SNF5, therefore affecting the transcription of genes involved in hormone signaling (Zraly & Dingwall, 2012). The maize SWI3D protein, ZmCHB101, impacts alternative splicing by influencing transcriptional elongation rate mediated by RNA Pol II in response to osmotic stress (Yu et al. 2019). Schwabish and Struhl (2007) reported that SWI/SNF affects RNA Pol II elongation thereby influencing splicing through two possible mechanisms, including the association with Pol II or factors that travel with elongating Pol II, and recognition of distorted chromatin that occur during transcriptional elongation process to permit passage of Pol II.

A widely reported mechanism of SWI/SNF controlling splicing is by recruitment of splicing machinery, e.g., spliceosomal components, splicing factors (SFs) and RNA binding proteins (RBPs). In fission yeast, SWI/SNF contributes to splicing catalysis by promoting the recruitment of Prp2 ATPase, which functions to destabilize SF3 immediately before the first step of catalysis (Patrick et al. 2015). Similarly, SWI/SNF is considered as a key regulator in *S. cerevisiae* meiotic splicing, because it can lead to the redistribution of spliceosomes from ribosomal protein genes (RPGs), where the splicing of RPGs can be finely tuned to the environmental conditions and nutrient availability encountered by cells (Pleiss et al. 2007; Venkataramanan et al. 2017). Human and *Drosophila* SWI/SNF family influences splicing when adapting to environmental stimuli via physical interaction and snRNPs U1/U5 recruitment (Batsché et al. 2006; Tyagi et al. 2009). Studies in HeLa and *Schizosaccharomyces pombe* cells both revealed that interactions between U2 snRNP and SWI/SNF subunits influence splicing process (Cavellán et al. 2006; Fair & Pleiss, 2017; Makarov et al. 2012; Patrick et al. 2015). Specifically, human SWI/SNF complex may serve as an adaptor for U2 snRNP association with chromatin. SWI/SNF proteins were identified as components of spliceosomal complex E (Makarov et al. 2012), and the overexpression of U2 snRNP components in ΔSWI/SNF cells led to inefficient splicing of many introns in fission yeast (Patrick et al. 2015). Moreover, studies in *Drosophila* indicated that SWI/SNF plays a role in pre-mRNA processing, possibly by modulating the recruitment and/or assembly of splicing factors (Waldholm et al. 2011). BAF57/SMARCE1 interacts with splicing factor SRSF1 to regulate mechanical stress-induced alternative splicing of cyclin D1 in osteoblast cells (Feng et al. 2021). In addition to splicing factors, recruitment of RNA binding proteins is also an important way to modulate splicing by SWI/SNF complex. For instance, in human cells, the ATPase Brm in concert with the mRNA-binding protein p54 regulate the splicing of telomerase reverse transcriptase (*TERT*) by accelerating exon-inclusion (Ito et al. 2008). Expression of the ATPase BRG1 in cervical cancer C33A cells promotes the local recruitment of splicing-RNA binding factors to chromatin and RNA, and further alters their binding to the nascent pre-mRNA, subsequently affecting alternative splicing (Zapater et al. 2019).

**Fig. 2:**
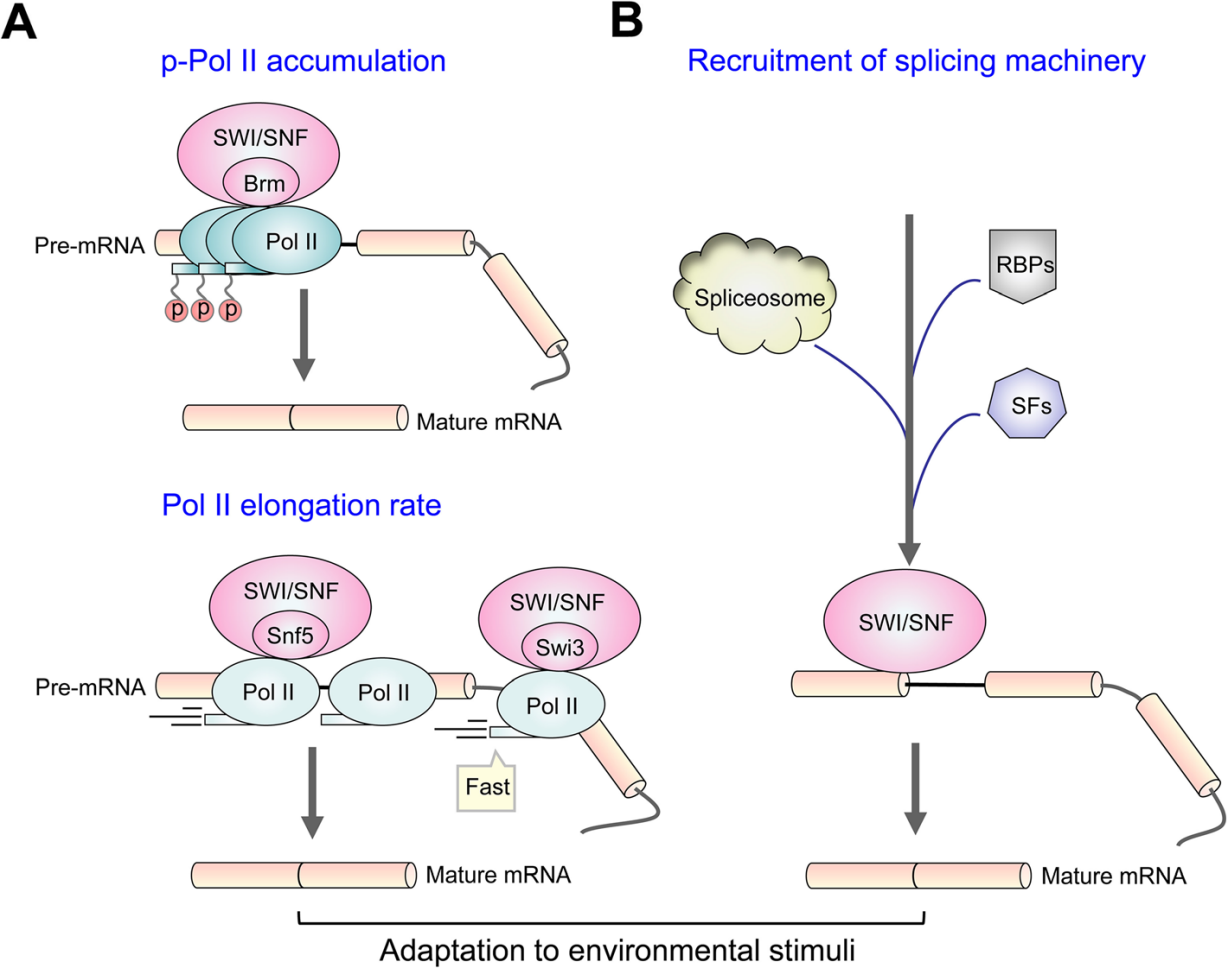
Two mechanisms by which SWI/SNF complex may affect splicing to adapt environmental stimuli. **A** The overexpression of Brm causes accumulation of phosphorylated RNA Pol II, thereby leading to increased mature mRNA. The core subunits of SWI/SNF complex SNF5 and Swi3, can modulate the transcription elongation rate of RNA Pol II at the target pre-mRNA, thus affecting the transcripts of hormone signaling and osmotic response, respectively. **B** SWI/SNF complex regulates pre-mRNA splicing by recruiting components of spliceosome, SFs (Splicing factors), as well as RBPs (RNA binding proteins) to target pre-mRNA. Both of the mechanisms promote splicing progress

Taken together, SWI/SNF complex influences splicing by two distinct but conserved mechanisms, including modulation of RNA Pol II accumulation/elongation and recruitment of splicing machinery (Allemand et al. 2008; Batsché et al. 2006; Kornblihtt, 2006) (Fig. 2). Moreover, the function of splicing in response to external stimuli is an ubiquitously accepted way that allows eukaryotes to quickly adjust the abundance of functional transcripts to environmental perturbations (Allemand et al. 2008; Capovilla et al. 2015; Pleiss et al. 2007). While studies in fungi remain limited, especially those in pathogenic fungi, summarizing SWI/SNF interactors affecting splicing events upon stress stimuli in plants and animals is important and provides significant reference for research in pathogenic fungi.

### Involvement of the SWI/SNF complex in DNA damage repair (DDR)

DNA damage can be corrected by DNA repair pathways (Ataian & Krebs, 2006; Lindahl, 2000). However, the genomic DNA packaged inside chromatin hinders DNA accessibility, and therefore the DNA repair and signaling machineries have to overcome this chromatin barrier to access the lesion (Ataian & Krebs, 2006; Smeenk & van Attikum, 2013; Wang et al. 2020). An increase in chromatin mobility has also been observed at lesions of budding yeast and human cells exposed to DNA damage (Hauer et al. 2017; Miné-Hattab & Rothstein, 2012; Roukos et al. 2013), which suggests a tight relationship between DNA damage repair (DDR) and chromatin structure.

**Fig. 3:**
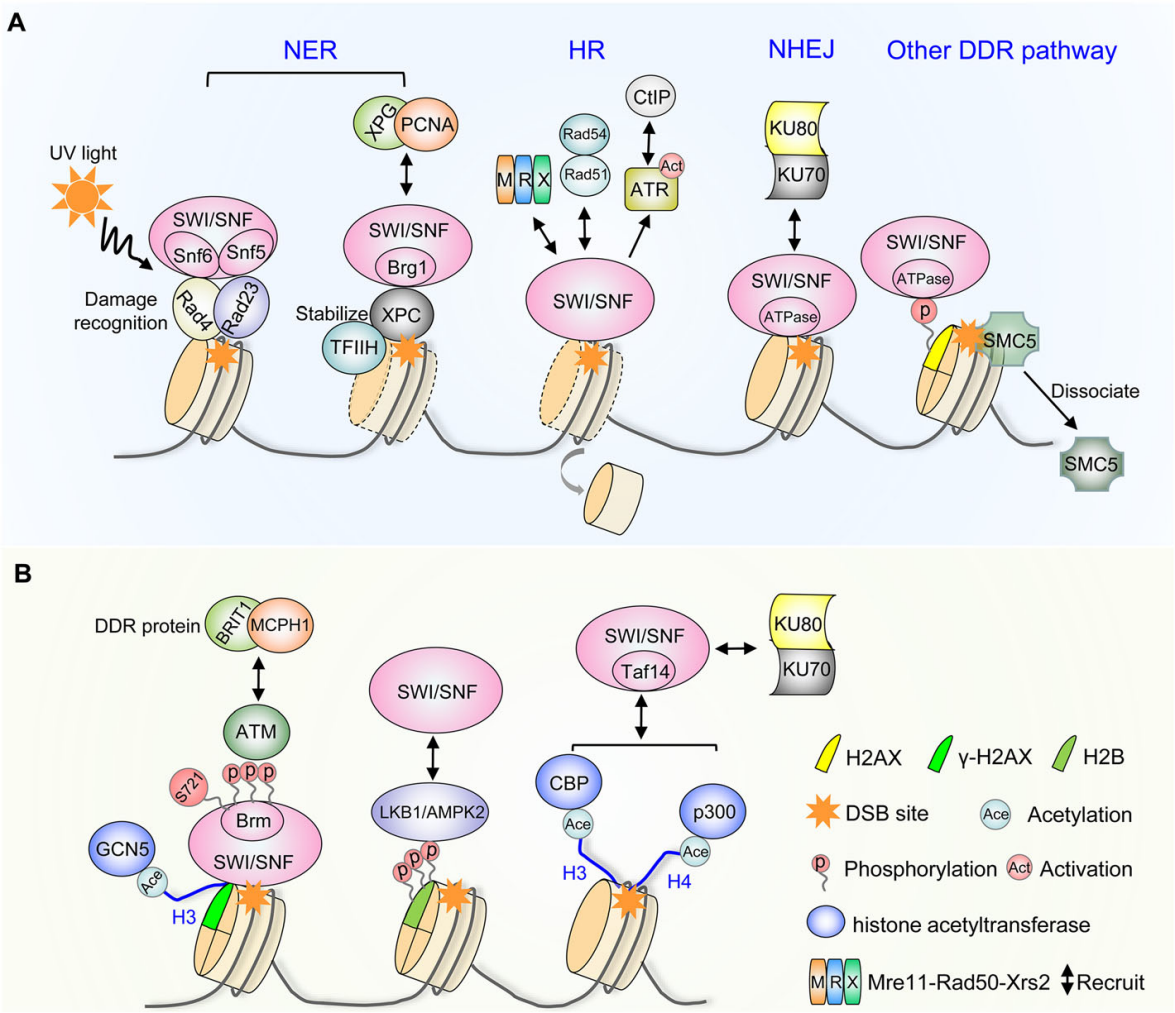
Regulatory mechanisms of the SWI/SNF complex in various DNA damage repair pathways. **A** Various models of how different subunits of SWI/SNF complex can recruit or be recruited by DNA damage associated factors to facilitate DNA damage repair (DDR) pathways, such as NER, HR, NHEJ and other DDR pathways. **B** Phosphorylation of ATPase or acetylation/phosphorylation of histone are implicated to promote the localization of SWI/SNF complex and further recruit DNA damage associated factors at the damage loci

ATP-dependent chromatin remodeling complex SWI/SNF members function directly in nucleotide-excision repair (NER), double-strand break (DSB) repair and other DDR pathways by modifying chromatin structure around DNA damage sites and further recruiting DDR proteins (Bao & Shen, 2007; Mittal & Roberts, 2020; Ribeiro-Silva et al. 2019; Smeenk & van Attikum, 2013) (Fig. 3). NER is a versatile DNA repair pathway that can remove a variety of structurally unrelated lesions including UV-induced bulky DNA adducts (Spivak, 2015). In *S. cerevisiae*, damage-recognition heterodimer Rad4-Rad23 associates with Snf6 and Snf5, two subunits of the SWI/SNF complex to increase DNA accessibility for NER in chromatin, and their association is stimulated by UV irradiation (Gong et al. 2006). Moreover, BRG1 facilitates NER at different stages by modulating chromatin relaxation and stabilizing xeroderma pigmentosum group C protein (XPC) at the damage sites, which subsequently promotes xeroderma pigmentosum group G protein (XPG), and proliferates cell nuclear antigen (PCNA) recruitment to complete the repair in mammalian cells (Zhao et al. 2009). Inactivation of ATPase subunits downregulates GTF2H1, a core subunit of the transcription factor IIH (TFIIH) complex, thus compromising TFIIH stability and NER pathway (Ribeiro-Silva et al. 2018).

In addition to NER, the SWI/SNF complex also functions in homologous recombination (HR) and non-homologous end-joining (NHEJ), which are two major conserved DSB repair pathways (Bao & Shen, 2007; Smeenk & van Attikum, 2013). For example, the chromatin remodeling activity of SWI/SNF contributes to Mre11-Rad50-Xrs2 (MRX) recruitment and resection initiation during HR, as nucleosome eviction at a DSB site is observed to be delayed in a SWI/SNF mutant (Wiest et al. 2017). Promoting the Rad51- and Rad54-dependent strand invasion during recombinational repair of the mating-type loci also requires SWI/SNF complex to alleviate heterochromatic constraints (Hauer & Gasser, 2017; Sinha et al. 2009). Consistently, downregulation of BRG1 and BRM in human cells reduced HR efficiency by 40–50% and 15%, respectively. BRG1 is important for activating ATM- and Rad3-related (ATR) kinase and reducing nucleosome density at DSBs, which further changes the chromatin structure and promotes CtIP nuclease recruitment, thus stimulating DNA end resection and HR (Hays et al. 2020). In both yeast and mammalian cells, BRM stimulates recruitment of NHEJ factors KU70/KU80, while BRG1 promotes HR-associated DNA end resection and RPA and RAD51 loading (Ribeiro-Silva et al. 2019). SWI/SNF complex also participates in other DDR pathways. For example, inactivation of ATPase subunits compromise H2AX phosphorylation, thereby affecting their function in DSB repair (Park et al. 2006). Meanwhile, the depletion BRG1 increases R-loops and R-loop-dependent DNA breaks as well as transcription-replication conflicts (Bayona-Feliu et al. 2021). In plant cells, the presence of an appropriate level of the SWI/SNF subunit SWI3B enhances the dissociation of structural maintenance complex 5 (SMC5) from chromosomes for its further recruitment at DSBs during DNA damage (Jiang et al. 2019).

Depletion, mutation or loss of SWI/SNF subunits has been shown to cause defects in DNA damage repair, suggesting that SWI/SNF complex is rapidly recruited to DSBs (Harrod et al. 2020). But how does SWI/SNF complex know when and where to find targets? Post-transcriptional modifications have been implicated to promote the localization of SWI/SNF to DNA damage sites. For instance, A-T mutated (ATM)-mediated phosphorylation of BRG1 and BAF170 increases the association of SWI/SNF complex with the early DDR protein BRIT1/MCPH1 (Kwon et al. 2015; Peng et al. 2009). The Ser-721 phosphorylation of BRG1 by ATM facilitates DSB repair by stimulating the association with γ-H2AX nucleosomes via enhancing the affinity to acetylated H3 (Kwon et al. 2015). The histone H2B kinase AMPK2, a major substrate of the tumor suppressor LKB1, is recruited to DSBs by LKB1. Disruption of the AMPK2 phosphorylation site impairs BRM recruitment to DSB sites, which further fails to activate NHEJ pathway (Ui et al. 2014). In addition, the acetylation of histone H3 and H4 has been shown to facilitate SWI/SNF recruitment. CBP and p300 acetylate H4 K5/K8/K12/K16 and H3 K18 to facilitate SWI/SNF chromatin remodeling at DSBs, which may provide access to the damage site for the NHEJ factors Ku70/Ku80 (Ogiwara et al. 2011). The SWI/SNF subunit Taf14 functions as a selective reader of histone H3 Lys9 acetylation (H3K9ac), and disruption of this binding in cells impairs the transcription of DNA damage response genes (Shanle et al. 2015). γH2AX recruits histone acetyltransferase GCN5 to trigger acetylation of H3, which subsequently attracts SWI/SNF binding (Lee et al. 2010; Park et al. 2006). Other mechanisms can also contribute to SWI/SNF recruitment at DSBs, for example, DDR protein BRIT1/MCPH1 associates with core subunits of SWI/SNF and promotes their recruitment and retention (Lukas et al. 2011; Peng et al. 2009). During methyl methanesulfonate (MMS) induction in *S. cerevisiae*, the activator Crt1 facilitates the recruitment of TFIID and SWI/SNF, which in turn promotes chromatin remodeling and preinitiation complex assembly (Ghosh & Pugh, 2011).

## Roles of the SWI/SNF complex in plant-pathogen interaction

As sessile organisms, plants must properly modulate their gene expression to survive in the environment, which is greatly influenced by the dynamic chromatin structure (Chang et al. 2020; Song et al. 2021). Mounting evidence has shown that SWI/SNF complex plays important roles in plant abiotic stress responses, including temperatures, drought, salt, osmotic stress and hormone signaling pathways as well as biotic stresses (Bhadouriya et al. 2020; Chang et al. 2020; Song et al. 2021; Thouly et al. 2020). In this review, we focus on deciphering the roles of this chromatin remodeling complex in response to plant biotic stress, which will advance our understanding in plant-pathogen interactions.

### The SWI/SNF complex regulates plant immune response

Unlike animals, plants lack an adaptive immune system that produces antibodies and lack mobile cells to detect, prevent or reduce infections once perceiving microbial pathogens. Instead, plants have evolved an innate immunity system to defend against microbial attack (Ding & Wang, 2015). Phytohormones including salicylic acid (SA), jasmonic acid (JA), ethylene (ET), abscisic acid (ABA), auxins, gibberellins (GA), cytokinins, and brassinosteroids, emerged as important players in plant immune response (Pieterse et al. 2009). Accumulating evidence has shown that chromatin structures play essential roles in regulating plant defense responses via physical association with the components or regulators of phytohormone signaling pathways (Chen et al. 2017; Ding & Wang, 2015; Ojolo et al. 2018; Ramirez-Prado et al. 2018; Sarnowska et al. 2016).

In Arabidopsis, BRM binds to abscisic acid (ABA)-induced basic domain/leucine zipper transcription factor ABA INSENSITIVE5 (ABI5) locus to stabilize the local nucleosome, thus negatively regulating its expression level in the absence of stress stimuli (Han et al. 2012). A two-hybrid analysis revealed that the core subunit of SWI/SNF complex SWI3B interacts with HYPERSENSITIVE to ABA1 (HAB1), a protein phosphatase type 2C, to modulate ABA signaling (Saez et al. 2008). In addition, the dysregulation of pathogenesis-related (PR) genes in the BRM101 mutant indicates a role for this protein in salicylic acid (SA)-mediated resistance (Bezhani et al. 2007; Chen et al. 2017; Han et al. 2012). BRM also directly associates with the promoters of GA biosynthesis genes to positively regulate its expression, whereas the loss of BRM leads to remarkable decrease level of endogenous bioactive GA (Archacki et al. 2013; Reyes, 2014). Moreover, several subunits of SWI/SNF complex (SWI3B, SWI3D, and BSH) interact with the miRNA-binding protein ARGONAUTE1 (AGO1) to facilitate its binding to chromatin upon JA, auxin, and SA stimuli in Arabidopsis (Liu et al. 2018a; Maury et al. 2019).

In addition to regulating phytohormone signaling pathways, the SWI/SNF complex plays important roles in modulating transcription and splicing of resistant genes. In Arabidopsis, SWP73A directly binds to the promoters of NLR (NOD-like receptor) genes to suppress their expression level. In addition, it may also function as a H3K9me2 reader to enhance this transcription suppression (Huang et al. 2021), as H3K9me2 is a transcriptional repression marker in plants (Pfluger & Wagner, 2007). Moreover, SWP73A affects the alternative splicing of some NLRs through indirectly suppressing the key regulator of RNA splicing CDC5, thus inhibiting the defense responses in Arabidopsis. In turn, bacteria-induced small RNAs silence SWP73A to activate a group of NLRs and trigger robust immune responses upon infection (Huang et al. 2021).

Other than the above mentioned subunits of SWI/SNF complex, another Arabidopsis ATPase, SYD, also plays important roles in plant defense against specific biotic stresses. Genetic, biochemical and biological evidences have shown that SYD is recruited to the promoters of genes controlling jasmonate (JA)- and ethylene (ET)-dependent defense responses to positively regulate their expression levels upon *Botrytis cinerea* infection (Walley et al. 2008). Moreover, SYD is required for resistance against the necrotrophic pathogen *B. cinerea* rather than the biotrophic pathogen *Pseudomonas syringae*, which indicates that chromatin remodeling participates in resistance to pathogens selectively (Walley et al. 2008). SNC1 (Suppressor of NPR1, Constitutive 1) is an intracellular Arabidopsis NLR protein that can be activated to induce defense responses (Zhang et al. 2003). Meanwhile, SYD functions antagonistically with MOS1 and MOS9 at the chromatin level to regulate SNC1 transcription and SNC1-mediated immunity (Johnson et al. 2015). Strikingly, the expression levels of SYD were found to be dramatically decreased upon indoleacetic acid (IAA), ABA, and benzothiadiazole (BTH) treatments and increased upon flg22 treatment, which suggests that SYD responds to pathogen attack at the transcription level (Shu et al. 2021).

In rice (*Oryza sativa* L.), the putative SWI/SNF2 class ATPase BRHIS1 is downregulated by the rice blast fungus *Magnaporthe oryzae* infection, suggesting that BRHIS1 plays a negative regulatory function in plant immunity (Li et al. 2015). RNA-seq and ChIP-seq data show that BRHIS1 suppresses the expression of a few defense-related genes (OsPBZc and OsSIRK1) rather than SA pathway genes, therefore regulating plant immunity in an SA-independent manner (Li et al. 2015). Further co-IP assays suggest that BRHIS1 specifically interacts with monoubiquitinated histone variants, H2A.Xa/H2A.Xb/H2A.3 and H2B.7 (Li et al. 2015). However, the enrichment of these histone variants at the promoter regions of OsPBZc and OsSIRK1 is correlated with their increased expression, whereas the BRHIS1 expression is suppressed (Li et al. 2015). These findings indicate that the BRHIS1 can relax chromatin state for defense genes by monoubiquitination local histone variants under normal growth conditions. Upon pathogen attack, BRHIS1 is inhibited, which makes chromatin accessible for defense gene expression. Together, the association between BRHIS1 and monoubiquitinated histone variant allows plants to establish an expression-ready chromatin state for defense genes to facilitate rapid activation of induced plant immune responses (Chen et al. 2017; Li et al. 2015).

### The signaling pathways regulates the SWI/SNF complex in plant

Although the pivotal role of SWI/SNF complex in plant immunity has been documented, how this complex is regulated in stress response or pathogen attack remains to be elucidated. One interesting study established a model suggesting that effector-SWI/SNF association plays vital roles during ectomycorrhizal-plant interactions. During infection, the effector-like protein PaMiSSP9.7 encoded by mycorrhizal fungus *Pisolithus albus* enters host root cells and localizes into the nucleus. Furthermore, it interacts with the SWI3D, a subunit of the SWI/SNF complex in host plant *Eucalyptus grandis*, that is activated and required to alter the outcome of mycorrhization (Aguirre, 2017).

More recently, Song and colleagues demonstrated the stability and activity of SWI/SNF subunits are also controlled by post-translational modifications (Song et al. 2021). Vicente and colleagues found that treatment with NaCl or ABA resulted in a decline in BRM protein (Vicente et al. 2017), which may be a result of the phosphorylation or dephosphorylation of BRM (Peirats-Llobet et al. 2016). Specifically, phosphorylation of BRM by the kinase SnRK2 contributes to its inhibition, whereas PP2CA-mediated dephosphorylation recovers the ability of BRM to negatively control ABA response (Peirats-Llobet et al. 2016). Another core subunit of SWI/SNF complex, SWI3B, physically interacts with the clade A PP2C phosphatase HAB1 (a core component in ABA signal pathway) and is directly dephosphorylated in an ABA-dependent manner (Saez et al. 2008). In addition to phosphorylation or dephosphorylation, the ubiquitin-proteasome system has also been shown to modulate BRM protein stability (Song et al. 2021). For instance, deletion mutants of *brm-3* and the SUMO ligase *mms21–1* both show resembling defects in root development, and the protein level of BRM is dramatically decreased in *mms21–1* mutant. Further biochemical evidence indicated that BRM is modified by SUMO3, and AtMMS21 enhances this SUMOylation to elevate BRM stability (Zhang et al. 2017). BRM is degraded by 26S proteasome in response to high-boron induced DSBs (Sakamoto et al. 2018), suggesting BRM is a target of 26S proteasome and is required for DSBs tolerance. During this process, BRM associates with histone acetylation to make chromatin more accessible, which could be inhibited by 26S proteasome (Sakamoto et al. 2018).

### The SWI/SNF complex plays important roles in fungal pathogenesis

During the infection process, pathogens encounter diverse stresses. To successfully invade hosts, fungi have evolved sophisticated strategies to adjust their developmental processes to adapt to the changes in various stimuli, including both the environment and the host (Łaźniewska et al. 2012). Several reports have demonstrated that the SWI/SNF complex is involved in stress responses and fungal pathogenesis (Table 2). The best characterized model is the most prevalent human fungal pathogen *C. albicans*. Knock-out mutation of SWI1 or SNF2, two core subunits of SWI/SNF complex, leads to a complete loss of pathogenicity in murine systemic candidiasis by inhibiting the expression of hyphae (filamentation)-specific genes (Mao et al. 2006). When cells encounter serum or nutrient starvation, the ATPase SNF2 is recruited by a synergistic action of NuA4 HAT complex and the hyphae-specific transcription factor Efg1 to the promoters of hyphae-specific genes, indicating that the SWI/SNF complex harnesses histone modifying enzyme to govern *Candidia* pathogenicity (Lu et al. 2008). Consistently, deletion of SNF5 leads to an altered metabolome and a loss of virulence, which implies its essential roles in maintaining metabolic homeostasis and pathogenicity in *C. albicans* (Burgain & Pic, 2019). The SWI/SNF complex is also involved in fluconazole resistance by cooperating with the inactivated form of transcription factor Mrr1 to promote nucleosome displacement from MDR1 (a multiple drug resistance gene 1) promoter, which further permits Mrr1 binding (Liu & Myers, 2017). Importantly, the fungal-specific subunit of SWI/SNF complex SNF6 is critical for *C. albicans* to colonize its host and to cause disease, suggesting SNF6 is a potential antifungal target (Tebbji et al. 2017). Similarly, in *Cryptococcus neoformans*, SWI/SNF assists transcription factor Znf2 to control yeast-to-hypha differentiation, which opens the promoter regions of hyphal specific genes, including the *ZNF2* gene itself, therefore facilitating fungal pathogenesis (Lin & Zhao, 2019).

In *Neurospora crassa*, white Collar-1 (WC-1) recruits SWI/SNF complex to remodel and loop chromatin at *FRQ*, thereby activating *FRQ* expression to initiate the circadian cycle (Wang et al. 2014). In *Trichoderma reesei*, the transcriptional activator of cellulase/hemicellulase genes, XYR1, interacts with the SNF12 subunit of SWI/SNF complex to remodel chromatin at cellulase gene promoters, thus activating their expression to initiate the cellulolytic response (Cao et al. 2019). In our previous studies, we found two transcription factors FgAreB and FgSR that recruit SWI/SNF complex via direct interaction with Swp73 to orchestrate genes responding to nitrosative and phytoalexin stresses, respectively, during *F. graminearum* infection (Jian et al. 2021; Liu et al. 2019). Importantly, Swp73 is essential in *F. graminearum* (Jian et al. 2021). These results are consistent with the cases observed in human, which also showed that BAF60a can serve as a bridge for the interactions between transcription factors and SWI/SNF complex (Iba et al. 2003; Oh et al. 2008). In addition, BAF60 (a Swp73 homolog) RNA interference mutant lines showed severe growth defects in Arabidopsis (Jégu et al. 2014), which further stressed the decisive role of this subunit across eukaryotes. Based on these published reports, we deduced that the disruption of interactions between BAF60 and transcription factors can cause transcriptional repression of a number of genes that participate in stress response.

It is widely accepted that reactive oxygen species (ROS) production by NADPH oxidases is one of the earliest responses of pathogen recognition in both plants and animals (Kadota et al. 2015). ROS acts as antimicrobials to prevent pathogen entry, and ROS-mediated DNA oxidative damage can be corrected by DNA repair pathways (Wang et al. 2020). Studies have established that the SWI/SNF complex is required for DNA damage repair, which is conserved from fungi to humans (Bao & Shen, 2007; Bohm et al. 2021; Harrod et al. 2020; Jiang et al. 2019; Ribeiro-Silva et al. 2019; Wiest et al. 2017). Therefore, we propose that the SWI/SNF complex plays a critical function in responses to ROS during plant-pathogen interactions. Although studies about the canonical SWI/SNF participates in this process have not been reported yet, another subtype of SWI/SNF family RSC has been characterized to modulate ROS response in phytopathogen-plant interactions. For example, silencing SFH1 (Snf5 homolog) in *Sclerotinia sclerotiorum* causes defects in hyphal growth and decreases ROS accumulation, suggesting its function in ROS production (Liu et al. 2018b). Also in the soil-borne pathogenic fungus *Verticillium dahlia*, VdDpb4 and VdIsw2 of ISWI chromatin remodeling complex play roles in maintaining chromatin structure for positioning nucleosomes and transcription regulation of DDR genes in response to ROS stress during plant infection (Wang et al. 2020).

**Fig. 4:**
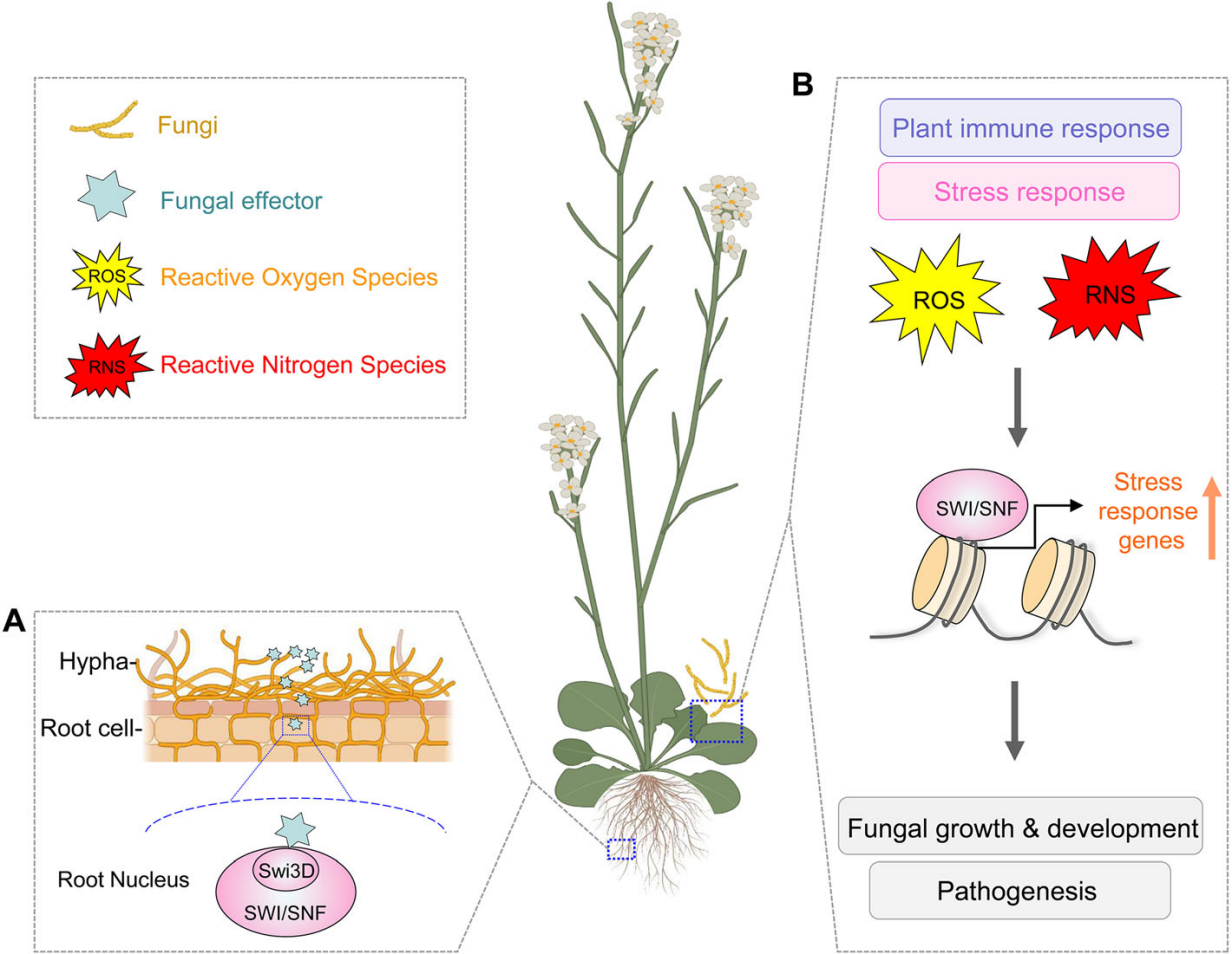
The SWI/SNF complex is involved in plant-pathogen interaction. **A** During *Eucalyptus grandis*-*Pisolithus albus* interaction, the effector-like protein PaMiSSP9.7 produced by the mycorrhizal fungus enters plant root nucleus to interact with Swi3D of SWI/SNF complex. **B** SWI/SNF complex mediates fungal development and pathogenesis by reprogramming the expression of stress responsive genes to phytohormone, ROS and RNS during various pathogen-plant interactions

In addition to ROS, host perception of pathogens can also provoke reactive nitrogen species (RNS), leading to nitrosative stress (NS) (Arasimowicz-Jelonek & Floryszak-Wieczorek, 2016). It is worth mentioning that our recent work suggests SWI/SNF complex can be recruited by transcription factor FgAreB to the nitrosative stress response (NSR) gene promoters*,* subsequently promoting NSR gene expression in *F. graminearum* (Jian et al. 2021). Furthermore, a transcriptional repressor, FgIxr1 was found to compete with the SWI/SNF complex to bind FgAreB, which negatively regulates NS response. In turn, NS promotes FgIxr1 degradation, therefore, enhancing the recruitment of the SWI/SNF complex by FgAreB (Jian et al. 2021). Taken together, SWI/SNF complex participates in plant-pathogen interaction via different routes (Fig. 4).

## Future perspectives

Nucleosomal structure is a barrier to transcription, DNA replication, and genome-wide DNA repair (Jansen & Verstrepen, 2011). SWI/SNF chromatin remodeling complex can utilize the energy derived from ATP hydrolysis to maintain proper nucleosome organization, thereby controlling major intracellular DNA-based biological processes (Becker & Hörz, 2002; Hohmann & Vakoc, 2014). Phylogenetic analyses and literatures suggest that the components of SWI/SNF complex and their mechanisms of operation are evolutionarily conserved across eukaryotes. However, studies of fungal SWI/SNF complex are limited, especially in phytopathogenic fungi, when compared to those in human and plants. Thus, summarizing and discussing the composition and function of SWI/SNF complex in higher eukaryotes will help advance our understanding of this complex in pathogenic fungi.

Genetic and biochemical experiments have revealed that the SWI/SNF complex is an essential regulator of numerous chromosomal processes, and its dysregulation leads to severe defects in development and stress response across eukaryotes (Clapier et al. 2017; Euskirchen et al. 2012; Jégu et al. 2014; Kwon & Wagner, 2007). This remodeler is built in a modular mode, with specific subunits that interact with or being regulated by specific activators/repressors/covalent histone modifiers or other functional proteins governing diverse stress responses. But how do these specific interactions provide such varied targeting repertoire, and how do they enable particular remodeler outcomes at specific locations? It will be of great interest to discover the SWI/SNF subunit interacting partners, combined with genetic, proteomics, transcriptomic and other high-throughput sequencing techniques, which will open new avenues to characterize a divergent set of fungal SWI/SNFs and their specific biological roles in pathogen-plant interaction. Further characterization of the SWI/SNF complex, including its organization, assembly, 3D structure, interaction partners, molecular regulatory mechanisms, and their roles in pathogen-plant interactions are expected to generate novel strategies that will help develop prevention measurements of plant diseases.

## Data Availability

Not applicable.
